# When does morbidity start? An analysis of changes in morbidity between 2013 and 2019 in Italy

**DOI:** 10.1007/s10260-022-00668-9

**Published:** 2022-10-24

**Authors:** Andrea Pastore, Stefano F. Tonellato, Emanuele Aliverti, Stefano Campostrini

**Affiliations:** Department of Economics, Ca’ Foscari University, Venice, Italy

**Keywords:** Health-statistics, Longevity, Morbidity compression, Surveillance system

## Abstract

Morbidity is one of the key aspects for assessing populations’ well-being. In particular, chronic diseases negatively affect the quality of life in the old age and the risk that more years added to lives are years of disability and illness. Novel analysis, interventions and policies are required to understand and potentially mitigate this issue. In this article, we focus on investigating whether in Italy the compression of morbidity is in act in the recent years, parallely to an increase of life expectancy. Our analysis rely on large repeated cross-sectional data from the national surveillance system passi, providing deep insights on the evolution of morbidity together with other socio-demographical variables. In addition, we investigate differences in morbidity across subgroups, focusing on disparities by gender, level of education and economic difficulties, and assessing the evolution of these differences across the period 2013–2019.

## Introduction

Over the years, longevity has increased in the entire world. Particularly in the western countries, the combination with a substantial decrease in fertility rates has brought to a continuous ageing of the population. In many countries, the elderly subgroups are already more numerous than the young ones, and old-age-dependency indicators—such as population older than 65 in retirement years over active population aged 18–65—are steadily increasing. In Italy, life expectancy at birth in 2019 (before Covid-19 pandemic) was 81.1 years for males and 85.4 years for females, while in 1990 it was 73.6 and 80.1, respectively (retrived at https://demo.istat.it/tvm2016). In addition, the age dependency ratio was 36.2% in 2020, while it was 21.7% in 1990.

For the sustainability of health and welfare systems, the only possible perspective to afford such an ageing in population is that of a compression of morbidity (Fries 2003; Ismail et al. 2016). Morbidity compression can be defined as a delay in the onset of morbidity with a combined effect of a reduction of the prevalence of chronic condition among the elderly population. In countries like Italy, in order to maintain a universally accessible health system, it is fundamental to have healthier elderly people.

Non-communicable-diseases (NCDs) are the main cause of morbidity in many countries (see, for instance, Monasta et al. (2019) for the Italian case), and are also defined as chronic, since most of them carry to a chronic condition that leads to higher levels of disability. For this reason, as known, NCDs determine an increasing use of health care resources (Kassebaum et al. 2016; Fitzmaurice et al. 2019). Indeed, morbidity compression is one of the key aspects in relation to populations’ longevity, but also, and even more relevantly, for elderlies’ quality of life (e.g., Chatterji et al. 2015; Fries et al. 2011). Despite the relevance of these issues, morbidity compression has not been so widely studied (Welsh et al. 2021; Newton 2021). In Italy, some works have been done recently using data from ISTAT Health Interview Survey conducted every five years, showing a possible increase of health condition among the elderly population (Demuru and Egidi 2016; Caselli et al. 2021).

In this article, we are interested in evaluating the evolution and the dynamic of the compression of morbidity in Italy in the latest years, focusing on the overall trend and on socio-demographic differences. Specifically, in order to understand possible changes in morbidity evolution among cohorts, we conducted several analyses on data coming from a NCDs surveillance system running since 2007 (Baldissera et al. 2011). The use of surveillance data instead of other official statistics has been motivated by the rapid availability of these kind of data combined with a continuous collection over time (Campostrini et al. 2015; Campostrini and McQueen 2005). This allows not only to detect whether a compression of morbidity is in act in Italy, but also to study how morbidity is changing over time according with age, sex, education level and economic status.

## Methods

### The passi surveillance system

Data come from the Italian surveillance system passi (https://www.epicentro.iss.it/passi/en/english), that measures changes in the major health risk factors among the adult population, in addition to several other health factors such as access to prevention services, self-declared health status diagnosed diseases and socio-demographic variables (see Baldissera et al. 2011, for more methodological issues, and Possenti et al. 2021, for a recent overview, as well as the official web site https://epicentro.iss.it/passi for further insights on how the surveillance system is carried out and on the validity of the indicators used). In passi, monthy cross-sectional samples are drawn from the lists of enrolled residents in each local health units, stratified on the basis of the most recent figures offered by istat by gender and three age groups (18–34, 35–49, 50–69 years). For this reason, the passi interviewees have substantially the same characteristics of the Italian adult population at the time of interview (Baldissera et al. 2011).

In this paper, morbidity is measured through a binary variable detecting the diagnosis of at least one among the following diseases:

- diabetes;
- kidney failure;
- chronic bronchitis, emphysema, respiratory failure;
- myocardial infarction, cardiac ischemia or coronary heart disease;
- tumors (including leukemias and lymphomas).

Such information is collected during the interview, asking if the physician has ever diagnosed each of the previous diseases.

A binary variable representing morbidity is a strong simplification, since it would be more appropriate to weight each disease condition considering its burden on disability. Given the purposes of this study, at this step aiming to test the approach and to find a suitable statistical methodology, we decided to accept this limitation, postponing the refinement of the model to a later time.

The diagnoses considered are those for which data are available for the entire period considered in this paper, from 2011 to 2019. Other diagnoses were introduced into the passi survey after 2011, and could be considered by limiting the analysis to a more recent period. Currently, the passi survey also includes information for the following additional diagnoses: bronchial asthma, stroke or cerebral ischemia, additional heart diseases, chronic liver disease, cirrhosis, arthrosis or arthritis; other diseases, such as mental or neurological disorders, are currently not included in the survey. In this paper, data from the passi survey are modeled and then combined with survivorship data produced by ISTAT. Therefore, similarly to what is done for the calculation of mortality rates in survival tables, passi survey data were also considered on a three-year basis, taking the last year of the three-year period as the reference. Therefore, estimates attributed to 2013 refer to the period 2011–13, those of 2016 to the period 2014–16, and those of 2019 to the period 2017–19.

We considered the following covariates for the morbidity analysis: year, age, gender, education level and a proxy of income, measured in terms of economic difficulties. The covariate year represents the reference period of the survey: in the data set we analyse it takes values 2013, 2016 and 2019. Age ranges between 40 and 69 years, since the prevalence of morbidity in young individuals is not relevant for the aims of this paper. Educational level has been simplified to a two–level categorical variable:

- low education level corresponds to levels 0, 1, 2 of 2011 ISCED (International Standard Classification of Education) classification (Unesco Institute for Statistic 2012);
- medium–high classification level corresponds to levels bewteen 3 and 8 of the 2011 ISCED classification.

The two level proxy of economic difficulties was set up from the following question: *Considering all the resources of your family, how do you get to the end of the month?*, where the possible answer were: *very easily, rather easily, with some difficulties, with a lot of difficulties*. The levels of the new variable were set up according to the following rule:

- yes when the answer was *with some difficulties* or *with a lot of difficulties*;
- no, otherwise.

The two-level simplification of the education level and economic difficulties covariates is motivated by the need to contain the number of model parameters and consequently to improve the precision of the obtained estimates.

**Table 1:** Illustrative subset of the dataset derived from the passi data

Year	Age	Economic difficulties	Education	Sex	$n_i$	$y_i$
2013	40	No	Low	M	2117	118
2013	41	Yes	Low	F	2233	143
2016	42	Yes	Low	M	2360	162
2016	43	No	Medium-high	M	2406	180
2016	44	No	Medium-high	F	2337	180
2013	45	Yes	Low	F	2486	194
2013	46	No	Medium-high	M	2494	207
2019	67	Yes	Medium-high	F	1863	591
2019	68	No	Low	M	1895	695
2019	69	Yes	Low	F	1624	617
$\vdots$	$\vdots$	$\vdots$	$\vdots$	$\vdots$	$\vdots$	$\vdots$

### The model

To address the main question of this study, we evaluate the effect of covariates and of their mutual interactions on morbidity, leveraging a Bayesian logistic regression model for grouped data. Specifically, we denote as $n_i$ the number of subjects within the *i*-th covariate combination, and as $y_i$ the number of individuals affected by morbidity. Table 1 depicts a subset of the data under analysis, for illustrative combinations of the available covariates.

We model the probability of morbidity $\pi _i$ that is, *the probability of being in a morbidity status* for the i-th combination of covariates, letting:

1 $$\begin{aligned}&(y_i \mid n_i, \pi _i) \sim \text{ Binomial }(n_i, \pi _i), \quad i = 1, \dots , I=720 \nonumber \\&\quad \text{ logit }(\pi _i) = \log \left( \frac{\pi _i}{1-\pi _i}\right) = {x}_i^T \beta \end{aligned}$$

where ${x}_i = (1,x _{i0}, \dots , x_{ip})$ denotes the vector of covariates (including an intercept term and interactions), $\beta =(\beta _1, \dots , \beta _p)$ denotes the vector of associated coefficients and *I* is the number of different covariate combinations.

We specify an improper uniform prior on the coefficient vector $\beta$. Such a prior distribution is justified by the fact that the posterior distribution has shown to be unaffected by different prior specifications, due to the large sample size of the survey. Computations have been carried out via Markov Chain Monte Carlo (MCMC), relying on the R package MCMCpack (Martin et al. 2011). A random walk Metropolis Hastings algorithm has been run for 100,000 iterations, after 5000 burnin iterations. The variance-covariance matrix of the proposal distribution is proportional to the large sample variance-covariance matrix of the maximum likelihood estimator, with a tuning factor equal to 0.6. Due to the strong autocorrelation, we needed to thin the MCMC output by subsampling the chain every 20 iterations in order to reach a satisfactory mixing. Therefore, we ended up with a sample of size 5000, drawn from the posterior distribution of $\beta$. Variable selection has been carried out by fitting the full model, i.e. the model encompassing all the covariates and their interactions, and excluding those variables for which the 95% credible interval of the corresponding coefficient included the zero value.

Once a sample from the posterior distribution $p(\beta \mid y)$ is obtained, it is straightforward to produce an analogous sample from the posterior distribution of the probability of morbidity, $\pi _i$, and of the odds–ratios of interest, $\eta _{i,j}$, by simply applying the corresponding transformations to the MCMC output:

2 $$\begin{aligned} \pi _i = \text{ logit}^{-1}(x_i^T\beta ) ,\quad \eta _{i,j} = {\frac{\pi _i}{1-\pi _i}}{\frac{1-\pi _j}{\pi _j}}, \quad i,j = 1,\dots , I. \end{aligned}$$

### Model assessment

We assess the fit of our model via posterior predictive checks. Specifically, the posterior predictive distribution is given by

3 $$\begin{aligned} \text{ p }(y_{new}\mid x_{new}, n_{new}, y) = \int \text{ p }(y_{new} \mid x_{new}, n_{new}, \beta ) p(\beta \mid y) \ d\beta , \end{aligned}$$

and a sample from this distribution can be easily obtained by simulating from (1) after plugging in the parameter values drawn from $p(\beta \mid y)$. In order to assess model adequacy, we compared the realized and the predictive discrepancies as in Gelman et al. (1996), defining model discrepancy as:

4 $$\begin{aligned} X^2(y;\beta ) = \sum _{i=1}^I \frac{(y_i-E(y_i \mid \beta ))^2}{Var(y_i \mid \beta )}. \end{aligned}$$

Since we draw the sample $\beta _j,\ j=1,\dots , 5000$, from the posterior distribution of $\beta$, we can also determine the realised discrepancies $X^2(y;\beta _j)$ by means of a straightforward substitution in (4). In so doing, we produce a posterior sample for the discrepancy of the model given the observed data. For each such $\beta _j$, we can also draw a value $y_j^{rep}$ from (1), keeping the number of cases at each covariate combination fixed at the values observed in the sample, and finally compute 5000 determinations of the predictive discrepancy, i.e. $X^2(y_j^{rep};\beta _j)$. We can then compute the posterior predictive *p*-value, i.e. the Monte Carlo estimate of $\alpha = P\left( X^2(y^{rep};\beta )>X^2(y;\beta )\right)$. Values close to zero of $\hat{\alpha }$ provide evidence of model inadequacy. In our case, we had $\hat{\alpha }= 0.2258$, which indicates a good model fitting.

**Fig. 1:**
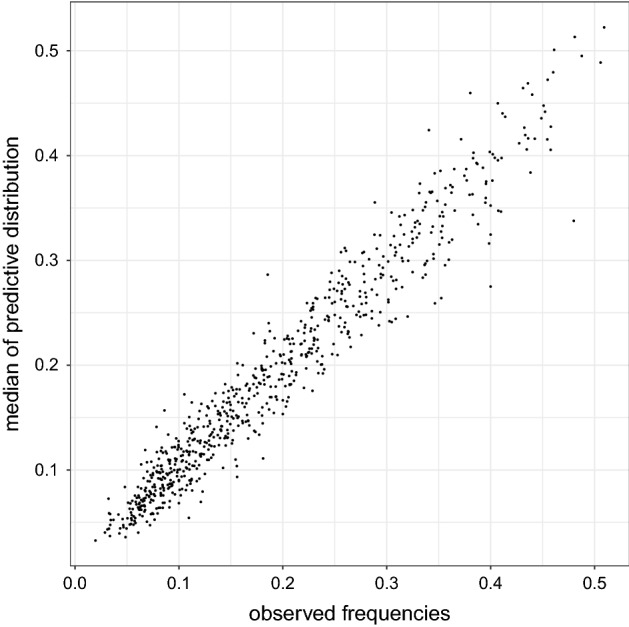
Observed versus predicted proportions for each of the 720 possible combination of the covariates’ value

In addition, we compared the estimated probabilities of morbidity via posterior medians with the observed proportions $y_i/n_i$, for each of the 720 different possible combinations of the covariates’ value. Figure 1 shows the scatterplot of observed and predicted proportions, suggesting a reasonably good fit.

## Results

**Table 2:** Summary statistics for the posterior distributions of the regression coefficients $\beta$ (mean, standard deviation and 95% credible intervals)

	Mean	SD	95% CI
(Intercept)	− 5.8189	0.0827	(− 5.9807, − 5.6569)
Year.2016	− 0.8664	0.1022	(− 1.0698, − 0.6645)
Year.2019	− 0.4649	0.1038	(− 0.6693, − 0.2584)
Age	0.0856	0.0014	(0.0828, 0.0884)
Female	1.3983	0.0862	(1.2337, 1.5671)
Educlev.H	− 0.2143	0.0129	(− 0.2397, − 0.1889)
Econdiff.N	− 0.3951	0.0127	(− 0.4200, − 0.3706)
Age:Female	− 0.0265	0.0015	(− 0.0295, − 0.0236)
Year.2016:age:Male	0.0120	0.0018	(0.0085, 0.0155)
Year.2019:age:Male	0.0052	0.0018	(0.0015, 0.0088)
Year.2016:age:Female	0.0115	0.0018	(0.0080, 0.0150)
Year.2019:age:Female	0.0060	0.0018	(0.0024, 0.0095)

The reference values for the non-quantitative covariates are: *year = 2013*, *sex = M*, *education = low*, *economic difficulties = no*. The parameter whose label includes a dot refers to the variable level specified after the same dot. The parameter whose label includes the colon, refer to an interaction term between the indicated levels or variables

**Fig. 2:**
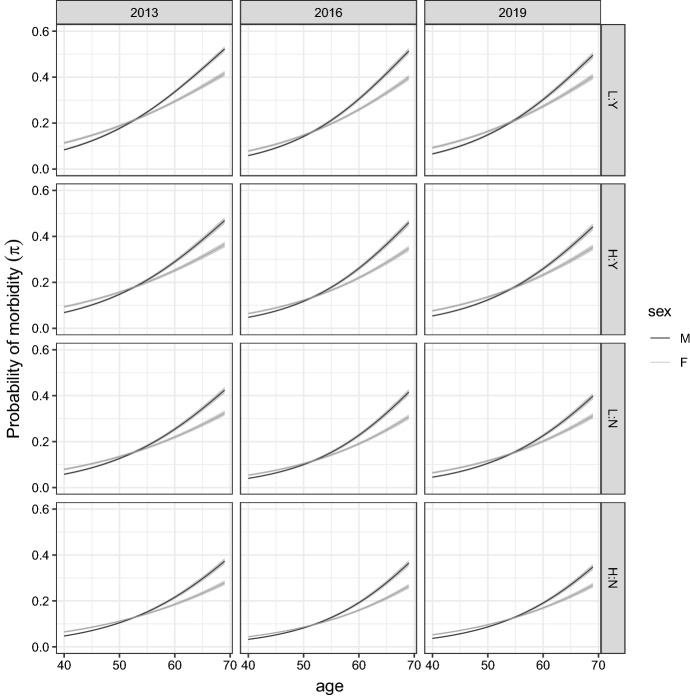
Posterior means and $95\%$ credible intervals for $\pi _i$ by age, year, and other covariates. Panels in each column refer to a given year, while panels in each row refer to a given combination of levels of the other two covariates *education* and *economic difficulties*. The labels for the rows are the following: H:N means *education = medium–high* and *economic difficulties = no*; L:N means *education = low* and *economic difficulties = no*; H:Y means *education = medium–high* and *economic difficulties = yes*; L:Y means *education = low* and *economic difficulties = yes*

A summary of the posterior distribution for the parameters is presented in Table 2. Our empirical findings indicate that a high level of education and the absence of economic difficulties both have a negative effect on morbidity, even controlling for the other covariates. However, the effect of the absence of economic difficulties is about twice as large as that associated with a high level of education. To assess the effect on morbidity of age, sex, and survey year, interactions between these variables must necessarily be taken into account.

These results are further explored in Fig. 2, where we conduct inference on the predicted probabilities $\pi _i$ provided by the model for all the possible profiles of the covariates. Each panel of Fig. 2 shows the posterior mean and $95\%$ credible intervals for the probability $\pi _i$ as a function of age, stratified by sex, year, education level and economic difficulties. Our empirical findings indicate a clear trend in terms of the probability of morbidity, which is increasing in age. In addition, as previously stated, individuals with high-educational level and without economic difficulties do experience on average a sensibly lower probability of morbidity at all ages; for example, in 2019 at the age 65 this value is less than 0.30 for men with high educational level and in a good socio-economic position (bottom-right panel of Fig. 2) whereas it is about 0.4 for males with low education and economic difficulties (top-right panel of the same figure). These findings confirm the existence of large differences in morbidity through socio-economic classes (Campostrini and McQueen 2014).

**Fig. 3:**
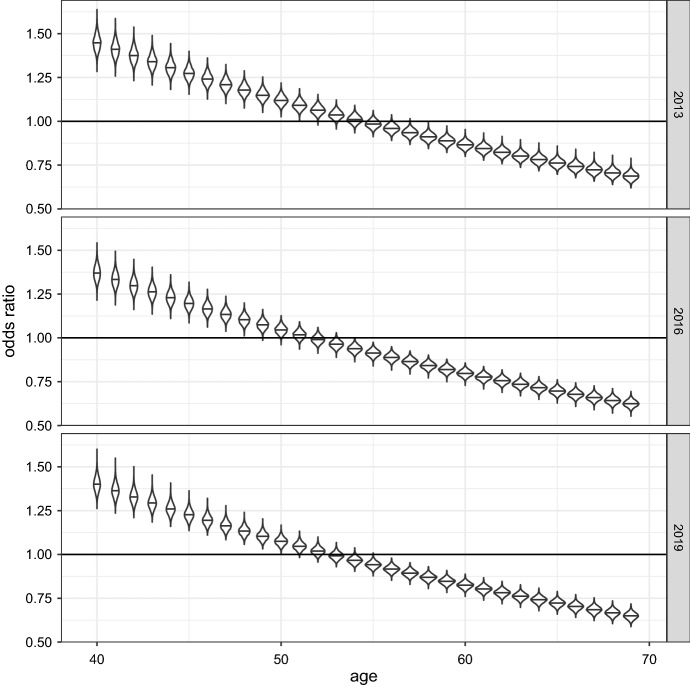
Violin plots of the posterior distribution of the odds ratio of gender (female/male), by age and year (the horizontal segment within the violin plots represents the median of the distribution)

Another interesting result concerns differences in morbidity by gender, which appear to be more likely among males than among females after a *crossover age* that is located, depending on the sampling year, between 50 and 55 years. In order to explore this aspect, we focus on the posterior distribution of the odds ratio of sex (female/male), for each combination of age and year; these posterior distributions are shown in Fig. 3, which gives a violin plot representation of the posterior probability density function of such odds ratios, where the horizontal segment within each violin plot represents the median of the distribution. By analyzing the content of this figure, it appears that males are in better conditions than females at younger ages, and that this advantage constantly decreases with age, and at the same time the dispersion of the odds ratio reduces considerably. We can estimate the crossover age relying on the odds ratio, considering the age at which the median of posterior distribution is closest to one. Such *crossover age* is 54 in 2013, 52 in 2016 and 54 in 2019, suggesting a stable trend in the window considered. Overall, current empirical findings confirm the trend of the *crossover age*, and suggest that improvements in morbidity have been more substantial across male population, in particular at older ages.

**Fig. 4:**
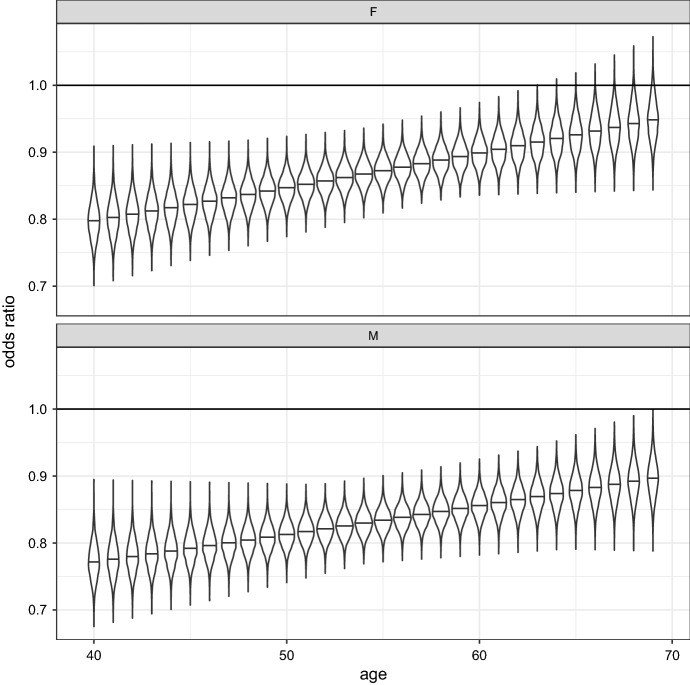
Violin plots of the posterior distribution of the odds ratio of year (2019 vs 2013), by age and sex (the horizontal segment within the violin plots represents the median of the distribution)

To assess how the risk morbidity has changed over the time period considered, odds ratios of the variable year (2019 vs 2013) were considered as a function of age and separately by sex. Figure 4 shows the violin plot representation of the distributions of such odds ratio (the horizontal segment within each violin plot represents the median of the distribution). The evidence is that for males there is a decrease in relative risk (thus an improvement in the “probability of morbidity” for all ages considered (40-69)). This also happens for females, but in this case we must be cautious in making assumptions about what happens at ages above 69.

**Fig. 5:**
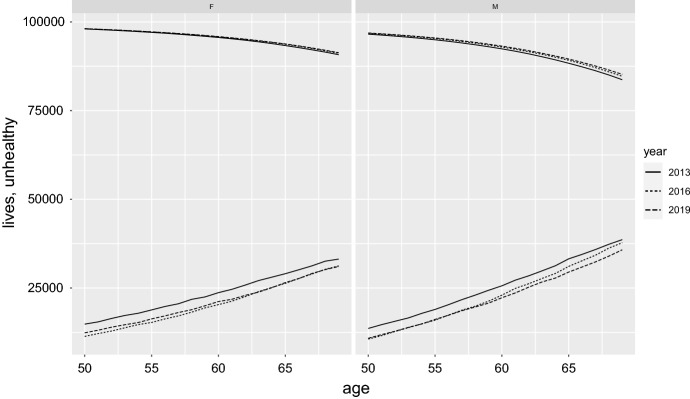
*Live* (source: Italian Statistical Institute) and *unhealty* population estimate (per 100,000 born), by age, sex and year. The *live* curves are the decreasing ones, at the top of the graphs, while the *unhealthy* curves are the increasing ones at the bottom

As a further utilization of data and models described above, we estimated the number of people living at a certain age in “unhealthy condition” (with at least one chronic disease), combining the posterior distribution of the probabilities $\pi _i$ and the survival curves for the Italian population (retrieved at https://demo.istat.it/tvm2016/). Figure 5 reports these estimates, for the considered sampling years (2013, 2016 and 2019), along with the number of people currently alive, both expressed per 100,000 born. Of course, the “live” curves are the decreasing ones, at the top of the graphs, while the “unhealthy” curves are the increasing ones at the bottom. For better readability, only ages between 50 and 69 are considered. The same information of Fig. 4 is shown, for some ages, in Table 3.

Comparing the three graphs, despite a limited but relevant increase in the number of people still living at the age of 69 (83,712 in 2013 and 85,223 in 2019, for Male, 90,762 in 2013 and 91,325, for Female), the number of people living in an unhealthy conditions is not increasing. It seems instead to be decreased of some hundreds over the 100,000 hypothetical cohort (38619 in 2013 and 35,756 in 2019, for Male, 33,144 in 2013 and 31053 for Female). These figures seem to confirm the hypothesis of a parallel process of increase in longevity and a compression of morbidity over the last decade in Italy. The combined effect seems to be re-assuring at least for the pre-elderly population (younger than 70). Further analyses and data are required in order to produce reliable inferences also for older population, offering in this way a complete scenario allowing us to judge the combined effect of longevity and morbidity over the years.

**Table 3:** *Live* (source: Italian Statistical Institute) and *unhealty* population estimate (per 100,000 born), by sex, years and some ages

Age	Sex	Lives (per 100,000 born)			Unhealthy (per 100,000 born)		
		2013	2016	2019	2013	2016	2019
50	M	96,545	96,791	96,888	13,599	10,597	10,818
50	F	98,015	98,086	98,106	14,811	11,352	12,357
55	M	94,963	95,325	95,479	18,954	15,934	16,149
55	F	97,050	97,179	97,212	18,812	15,283	16,283
60	M	92,410	92,907	93,175	25,612	22,977	22,251
60	F	95,613	95,789	95,871	23,679	20,306	21,172
65	M	88,346	89,172	89,530	33,221	31,093	29,489
65	F	93,342	93,713	93,798	29,021	26,303	26,509
69	M	83,712	84,683	85,223	38,619	37,759	35,756
69	F	90,762	91,165	91,325	33,144	31,229	31,053

## Discussion

Future scenarios for public health require necessarily estimates on morbidity trends, capable of showing if and how a morbidity compression is going along the clear (so far) increase in longevity. In this article, we have partially addressed this aim via a Bayesian logistic regression model for repeated cross-sectional survey data collected by the passi surveillance system. This model allows to estimate the relationship between age and the probability of being affected by at least on chronic disease, highlighting changes occurred in the past years and across sub-populations of interest. Acknowledging all the possible limits coming from observational studies based on respondents’ self-declarations, the richness of a surveillance system running for more than 12 years allows to, at least, propose some first tentative hypotheses. Another relevant limitation derives from our data source that limits the observation to the age of 69. Although the “pre-elderly” subgroup is perhaps the most relevant both for studying morbidity compression and to be targeted by preventive policies, this does not allow to observe and estimate the morbidity compression on all the country’s population.

From the first analyses it seems that a morbidity compression in Italy is acting in the last decade, noting a relevant double effect: postponing the average year in which the presence of a chronic disease starts, and reducing the prevalence of chronic diseases in pre-elderly age (40-69). Notably, the focus on this age group, rather than in the more usual elderly age (over 65) is relevant from a public health perspective: “successful aging” is built in earlier times (Hareven 1994; Stowe and Cooney 2015; Ory et al. 2003). Moreover, health promotion can prepare individuals and communities for healthier behaviors and an active aging, targeting specifically this age group.

In the period of observation, morbidity compression seems more relevant for men than for women, confirming the recent trends in a relative more consistent increase in longevity for males (e.g., Rosella et al. 2016; Sundberg et al. 2018). It is interesting to note how these are substantially different processes as can be easily detected observing the curves reported in Fig. 2. Italian male population has successfully postponed the age “at which morbidity starts”, gaining in only six years (2013–2019) more than two years “without morbidity”, as can be estimated (data not shown) considering the maximum accelaration point of the curves. Nevertheless, the estimation of the number of persons with at least one chronic disease at the age of 69 has not changed substantially over the years of observation as presented in the previous paragraph. On the other hand, females present a rather different pattern of the increase of morbidity over the ages. This process is much smoother and this brings to a much lower number of women with morbidity at the age of 70, in any year of observation. Although apparently not so consistent, the gain in lowering morbidity among females is steady at every age when comparing 2013 and 2016: the curve of morbidity over ages in 2013 is, at any point, above the one referred to 2019. These are comforting signals: as stated before, the morbidity compression seems to balance the longevity increase, also at the age here considered, maintaining almost constant the number of persons facing the elderly time in unhealthy (chronic) conditions.

Although trends appear positive, our study suggests also some relevant aspects that should be considered for future policies. The first one is about health inequalities: our analyses show how remarkable are the differences among population subgroups and, even more remarkably, how trends (in morbidity compression) seems not going forward “closing the gap”.

The second aspects is related to the demographic structure of the Italian population (similar, anyway, to that of several other western countries). The continue decrease in fertility will soon bring the weight of the elderly care on less and less shoulders. So, if the morbidity compression seems to balance the increase in longevity, at the level here estimated will not be able in the future to balance the steady increase in dependency indexes. This further motivate investments in public health, health prevention and promotion to enhance the health of the pre-elderly and elderly population.

As last remarks, trying to link substantial (public health) and methodological (statistical) aspects, our work seems to open promising perspectives.

The use of not necessarily novel, still sufficiently “sophisticated” statistical models on routinely available data, such as those coming from surveillance systems, allows to study such relevant issues as the one here addressed. And this not only to simply verify whether a morbidity compression is present, but also to better understand its mechanisms and how it is operating among population subgroups. Much research is still needed, also on the data already available: for instance, refining the indicator of morbidity and analyzing trends in population subgroups, trying to better understand the reason for which morbidity over age is so different among these groups.

Lastly, it is well known that major differences both in morbidity and mortality are present through socio-economic classes (Minardi et al. 2011; Campostrini and McQueen 2014); although our work has partially confirmed this, showing substantial differences in morbidity compression among more educated and relatively richer Italians, further analyses are needed to better explore the reasons for these differences. Anyway, also these first analyses provide enough evidence to support the need for better targeted interventions, also in countries like Italy that can rely on universal health systems, to tackle health disparities and meet the 2030 sustainable development goals number 3 and 8 (good health for all and reduced inequalities).
